# Genotype identification and phylogenetic analysis of *Enterocytozoon bieneusi* in farmed black goats (*Capra hircus*) from China’s Hainan Province

**DOI:** 10.1051/parasite/2019064

**Published:** 2019-10-31

**Authors:** Huan-Huan Zhou, Xin-Li Zheng, Tian-Ming Ma, Meng Qi, Zong-Xi Cao, Zhe Chao, Li-Min Wei, Quan-Wei Liu, Rui-Ping Sun, Feng Wang, Yan Zhang, Gang Lu, Wei Zhao

**Affiliations:** 1 Department of Pathogenic Biology, Hainan Medical University Xueyuan Road 3 571199 Haikou Hainan PR China; 2 Hainan Medical University-The University of Hong Kong Joint Laboratory of Tropical Infectious Diseases, Hainan Medical University 571199 Haikou Hainan PR China; 3 Key Laboratory of Tropical Translational Medicine of Ministry of Education, Hainan Medical University 571199 Haikou PR China; 4 Institute of Animal Science and Veterinary Medicine, Hainan Academy of Agricultural Sciences 571100 Haikou PR China; 5 College of Animal Sciences, Tarim University 843300 Alar Xinjiang PR China

**Keywords:** *Enterocytozoon bieneusi*, Genotype, ITS region, Goats, China

## Abstract

*Enterocytozoon bieneusi* is an important pathogen commonly found in humans and animals. Farmed animals with close contact to humans are important hosts of *E. bieneusi*. The role of goats in the transmission of *E. bieneusi*, however, remains unclear. In this study, 341 fresh fecal samples of black goats were collected from five locations in Hainan Province, China. *Enterocytozoon bieneusi* was identified and genotyped by sequences of the internal transcribed spacer (ITS) region. Phylogenetic analysis was performed by constructing a neighbor-joining tree of the ITS gene sequences. The average prevalence of *E. bieneusi* in black goats was 24.0% (82/341) with rates ranging from 6.3% (4/63) to 37.2% (32/86) across the locations (*χ*^2^ = 17.252, *p* < 0.01). Eight genotypes of *E. bieneusi* were identified, including six known genotypes: CHG5 (*n* = 47); CHG3 (*n* = 23); CHG2 (*n* = 4); CM21 (*n* = 3); D (*n* = 2); and AHG1 (*n* = 1), and two novel genotypes termed HNG-I (*n* = 1) and HNG-II (*n* = 1). In the phylogenetic tree, genotype D was clustered into Group 1 and the other identified genotypes were included in Group 2. This represents the first report identifying *E. bieneusi* in black goats from Hainan Province, with a high prevalence and wide occurrence demonstrated. The two new genotypes identified provide additional insights into the genotypic variations in *E. bieneusi*. Due to the small percentage of zoonotic genotypes in these animals, there is minimal risk of zoonotic transmission of *E. bieneusi*.

## Abstract (fr)

*Enterocytozoon bieneusi* est un agent pathogène important que l’on trouve couramment chez l’homme et les animaux. Les animaux d’élevage, en contact étroit avec l’homme, sont des hôtes importants d’*E. bieneusi*. Le rôle des chèvres dans la transmission d’*E. bieneusi* reste toutefois incertain. Dans cette étude, 341 échantillons de fèces fraîches de chèvres noires ont été prélevés dans cinq sites de la province de Hainan, en Chine. *Enterocytozoon bieneusi* a été identifié et génotypé par des séquences de la région de l’espaceur interne transcrit (ITS). L’analyse phylogénétique a été réalisée en construisant un arbre de jonction voisine des séquences du gène ITS. La prévalence moyenne d’*E. bieneusi* chez les chèvres noires était de 24,0 % (82/341), avec des taux allant de 6,3 % (4/63) à 37,2 % (32/86) dans tous les sites (*χ*^2^ = 17,252, *p* < 0,01). Huit génotypes d’*E. bieneusi* ont été identifiés, dont six génotypes connus: CHG5 (*n* = 47) ; CHG3 (*n* = 23) ; CHG2 (*n* = 4) ; CM21 (*n* = 3) ; D (*n* = 2) ; AHG1 (*n* = 1) et deux nouveaux génotypes appelés HNG-I (*n* = 1) et HNG-II (*n* = 1). Dans l’arbre phylogénétique, le génotype D appartenait au groupe 1 et les autres génotypes identifiés étaient inclus dans le groupe 2. Il s’agit du premier rapport identifiant *E. bieneusi* chez des chèvres noires de la province de Hainan, avec une prévalence élevée et une occurrence étendue. Les deux nouveaux génotypes identifiés fournissent des informations supplémentaires sur les variations génotypiques chez *E. bieneusi*. En raison du faible pourcentage de génotypes zoonotiques chez ces animaux, le risque de transmission zoonotique d’*E. bieneusi* est minime.

## Introduction

*Enterocytozoon bieneusi* is one of the most common species of microsporidia contributing to human microsporidiosis, which generally causes digestive disorders, including diarrhea, particularly in the young and in adults with immunodeficiency [17]. *E. bieneusi* is frequently found in numerous animal hosts worldwide, raising public health concerns regarding its zoonotic transmission [20]. The primary mode of infection by *E. bieneusi* is the fecal-oral route. *E. bieneusi* spores can be acquired from infected humans or animals through contaminated food and water [6]*.* Tracing the sources of contamination and elucidating the transmission routes of *E. bieneusi* represent important steps to control *E. bieneusi* infection in humans.

Sequence analysis of the ribosomal internal transcribed spacer (ITS) region is the standard method for the detection and identification of *E. bieneusi* genotypes [19]. To date, more than 500 genotypes have been identified, of which 130 have been found in humans [7, 13, 15, 26]. The known genotypes of *E. bieneusi* were divided into 11 phylogenetic groups (Groups 1–11) by phylogenetic analysis. Up to 90% of human pathogenic genotypes belong to Group 1 or Group 2, and the genotypes within these groups have been reported in a range of diverse hosts, highlighting the zoonotic nature of the disease and cross-species infections [13]. However, the genotypes in Groups 3–11 appear more amenable to host adaptation [13]. The contribution of each animal source to human infections is poorly understood. This can be clarified by the genotyping of *E. bieneusi* in different animals.

The goat industry plays a dominant role in animal husbandry in China. In recent years, a number of studies of *E. bieneusi* have been performed in goats, particularly those in China [1, 3, 4, 14, 16, 18, 21, 22, 28]. A total of 46 ITS genotypes of *E. bieneusi* have been identified in goats, 11 of which have been detected in humans [1, 3, 4, 13, 14, 16, 18, 21, 22, 28]. Nevertheless, the genotypes of *E. bieneusi* in goats in China are not fully understood.

Hainan black goats are native to Hainan, China and represent a common breed due to their tolerance to local hot and wet weather. They are the main goat breed in Hainan, China, where the goat industry represents an important source of poverty alleviation [8]. These animals are often in close contact with keepers, and their feces are commonly excreted directly into the surrounding environment without treatment. Environmental contamination with *E. bieneusi* spores represents a threat to public health, but no information on *E. bieneusi* in these animals is available. The aims of this study were to examine the prevalence and genotyping of *E. bieneusi* from farmed black goats in Hainan Province, the southernmost region of China.

## Materials and methods

### Collection of fecal specimens

**Figure 1:**
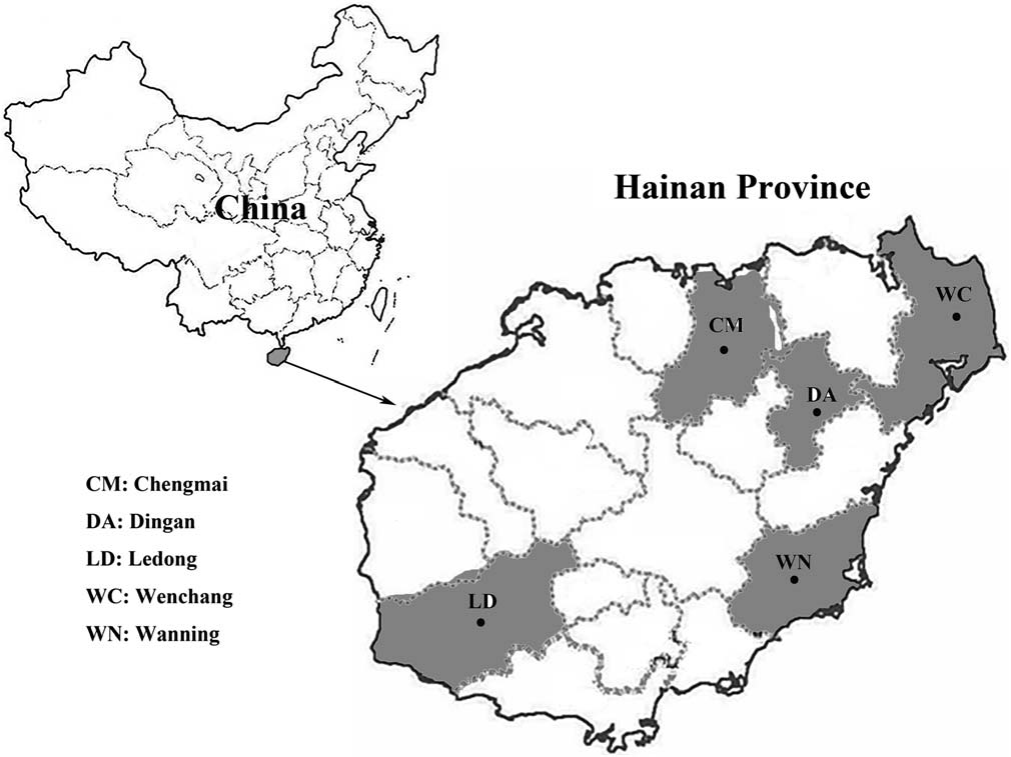
Specific locations where samples were collected in this study. ● Sampling points.

A total of 341 fresh fecal specimens from black goats were collected from Ding’an (*n* = 52), Chengmai (*n* = 63), Wenchang (*n* = 63), Ledong (*n* = 77), and Wanning (*n* = 86) in Hainan Province from September 2018 to March 2019 (Fig. 1). Sampled goats belonged to two groups: the first consisting of 73 animals aged ≤ 3 months (kids) and the other group consisting of 268 animals aged ≥ 4 months (older goats). The farms were selected based only on the owners’ willingness to participate and the accessibility of animals for sampling. All the goats were maintained in pens on the farms. Fecal samples were collected from pens housing 3–6 goats. To avoid the chance of duplicate sampling of animals, only one fecal specimen was collected in each pen. All the fecal specimens were collected at the bed boards immediately after defecation by using a sterile disposable latex glove and then placed in labeled sterile tubes individually. All bags were transported to our laboratory in a cooler with ice packs (<24 h) and stored at −20 °C until processing (<1 w).

### DNA extraction

All fecal specimens were sieved through an 8.0-cm-diameter sieve with a pore size of 45 μm, and filtrates were concentrated by centrifugation at 1500 *g* for 10 min. DNA was extracted from ~200 mg of each processed fecal specimen using a QIAamp DNA stool mini kit (QIAgen, Hilden, Germany). Extracted DNA was stored at −20 °C prior to PCR analysis.

### PCR amplification

Nested PCRs were performed to amplify a ~390-bp region containing 76 bp of the 3″ end of the SSU rRNA gene, 243 bp of the internal gene, and 70 bp of the 5′ region of the large-subunit (LSU) rRNA gene. The aim was to detect the presence of *E. bieneusi* in all the extracted DNA samples using two pairs of primers, EBITS3 and EBITS4, and EBITS1 and EBITS2.4 for the first and the second amplifications, respectively. The primers and cycling parameters were designed by Buckholt et al., as follows: the outer primers were EBITS3 (5′–GGTCATAGGGATGAAGAG–3′) and EBITS4 (5′–TTCGAGTTCTTTCGCGCTC–3′) and the inner primers were EBITS1 (5′–GCTCTGAATATCTATGGCT–3′) and EBITS2.4 (5′–ATCGCCGACGGATCCAAGTG–3′). The two sets of cycling parameters were 35 cycles of 94 °C for 30 s, 57 °C for 30 s, and 72 °C for 40 s and 30 cycles of 94 °C for 30 s, 55 °C for 30 s, and 72 °C for 40 s, with both of them having a final extension step at 72 °C for 10 min. These reactions produced fragments of 435 and 390 bp, respectively [2]. TaKaRa Taq DNA Polymerase (TaKaRa Bio Inc., Tokyo, Japan) was used for all PCR amplifications. A negative control with no DNA was amplified in all PCR tests, including the amplification of negative controls from the first PCR in the second PCR reaction, to ensure low levels of contamination. All secondary PCR products were analyzed on 1.5% agarose gels and visualized by GelRed staining (Biotium Inc., Hayward, CA, USA).

### Nucleotide sequencing and analysis

All secondary PCR products positive for *E. bieneusi* were sent for sequencing (Sangon Biotech Co., Ltd., Shanghai, China) and all were sequenced in both directions. The nucleotide sequences obtained were aligned with each other and reference sequences downloaded from the GenBank database using Clustal X 1.83 (http://www.clustal.org/) to determine the genotypes of *E. bieneusi* isolates. The genotypes of *E. bieneusi* obtained in this study were given the first published name when identical to known genotypes in GenBank. Meanwhile, the genotypes that produced ITS sequences with single nucleotide substitutions, deletions, or insertions confirmed by DNA sequencing of at least two PCR products were considered novel genotypes. All were given a genotype name through the addition of roman numbers behind the abbreviation HNG (Hainan Goat), according to their order of appearance. All genotypes were named based on a 243 bp region of the ITS gene of *E. bieneusi* according to the established nomenclature system [20].

### Phylogenetic analysis

To confirm the genogroup designation and to assess the genetic relationships of novel ITS genotypes of *E. bieneusi* obtained, phylogenetic analysis was performed by constructing a neighbor-joining tree using the program Mega X (http://www.megasoftware.net/) based on the evolutionary distances calculated by the Kimura-2-parameter model. The reliability of these trees was assessed using bootstrap analysis with 1000 replicates.

### Statistical analysis

Data entry and analysis were performed using Social Sciences (SPSS) 19.0 software. The statistical significance of differences in infection proportions was generally evaluated by Pearson’s Chi-square test. The significant level of all tests was: *p*-value = 0.05.

### Nucleotide sequence accession numbers

Representative nucleotide sequences obtained in the study were deposited in the GenBank database under accession numbers MN267058 and MN267059.

## Results

### Occurrence of *E. bieneusi* in black goats

A total of 24.0% (82/341) of black goat samples were positive for *E. bieneusi* by PCR and sequencing analysis. *E. bieneusi* was identified in all five locations with the highest infection rate of 37.2% (32/86) in Wanning, followed by 36.5% (19/52) in Ding’an, 30.2% (19/63) in Chengmai, 10.4% (8/77) in Ledong, and the lowest infection rate of 6.3% (4/63) in Wenchang. There were significant differences in prevalence among these locations (*χ*^2^ = 17.252, *p* < 0.01). The prevalence rates were 38.4% (28/73) and 20.1% (54/268) in kids and older goats, respectively, suggesting significant differences between them (*χ*^2^ = 10.413, *p* < 0.01) (Table 1).

**Table 1:** Prevalence and genotype distribution of *E. bieneusi* isolates from goats farmed in different cities in Hainan Province.

Factor	Category	Positive/examined (%)	Genotype/s (*n*)
Location	Chengmai	19/63 (30.2)	CHG5 (17), CHG3 (2)
	Dingan	19/52 (36.5)	CHG5 (11), CHG3 (6), CM21 (1), HNG-I (1)
	Ledong	8/77 (10.4)	CHG3 (8)
	Wanning	32/86 (37.2)	CHG5 (19), CHG3 (7), CHG2 (4), CM21 (2)
	Wenchang	4/63 (6.3)	D (2), AHG1 (1), HNG-II (1)
Age	≤4 month	28/73 (38.4)	CHG5 (20), CHG2 (3), CHG3 (2), CM21 (2), AHG1 (1)
	>4 month	54/268 (20.1)	CHG5 (27), CHG3 (21), D (2), CM21 (1), CHG2 (1), HNG-I (1), HNG-II (1)
Total		24.0 (82/341)	CHG5 (47), CHG3 (23), CHG2 (4), CM21 (3), D (2), AHG1 (1), HNG-I (1), HNG-II (1)

### Genetic characterization and genotypic distribution of *E. bieneusi* in black goats

**Figure 2:**
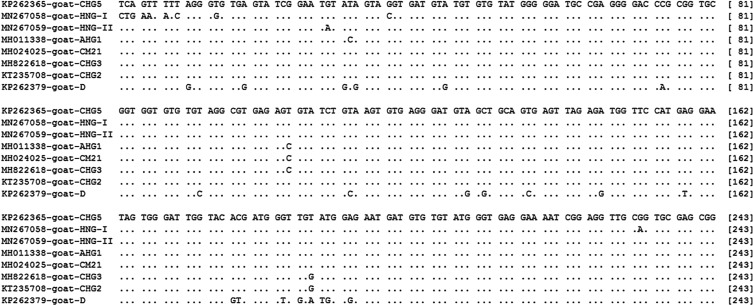
Sequence variation in the ITS region of the rRNA gene of *E. bieneusi* isolates identified in black goats. Dots indicate the same base identity as the ITS gene sequence of genotype CHG5.

Sequence analysis demonstrated that the 82 *E. bieneusi* isolates belonged to eight ITS genotypes including six known genotypes (AHG1, CHG2, CHG3, CHG5, CM21 and D), and two novel genotypes (HNG-I and HNG-II). There were 34 polymorphic sites among the eight genotypes identified (Fig. 2). The novel genotypes HNG-I (MN267058) and HNG-II (MN267059) had the largest similarity with genotype CHG5 (KP262365) with 10 and with one base difference, respectively. Among the genotypes, CHG5 (57.3%, 47/82) dominated, followed by CHG3 (28.0%, 23/82), CHG2 (4.9%, 4/82), CM21 (3.7%, 3/82), D (2.4%, 2/82), and each of the remaining three genotypes AHG1, HNG-I and HNG-II (1.2%, 1/82) (Table 1).

The distributions of *E. bieneusi* genotypes in animals according to location and age are shown in Table 1. Genotypes D, AHG1 and HNG-II were only found in Wenchang, while genotypes HNG-I and CHG2 were only present in Ding’an and Wanning, respectively. However, genotypes CHG3, CHG5 and CM21 were found in four, three and two areas, respectively. Regarding the age groups, genotypes CHG2, CHG3, CHG5, and CM21 were found in two age groups, while genotypes D, HNG-I and HNG-II were only found in older goats; genotype AHG1 was only found in kids.

### Phylogenetic relationships of *E. bieneusi* genotypes

**Figure 3:**
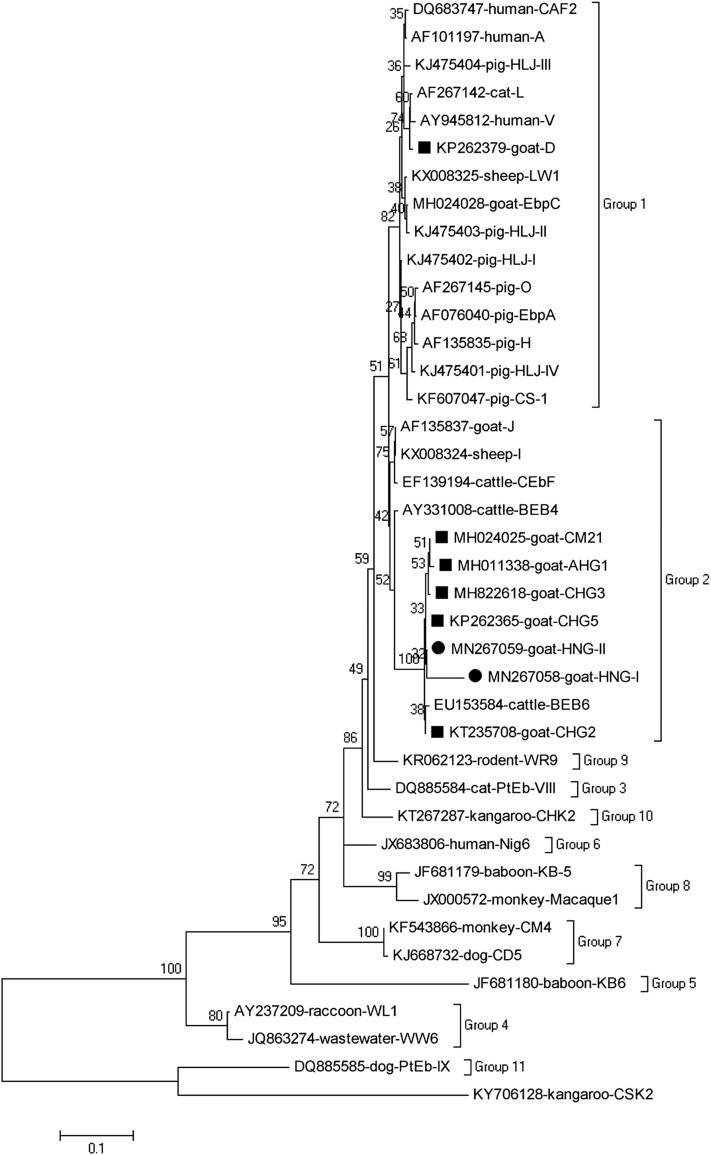
Phylogenetic tree based on the neighbor-joining analysis of ITS sequences. The phylogenetic relationships of *E. bieneusi* genotypes identified here and other known genotypes deposited in GenBank were inferred by a neighbor-joining analysis of ITS sequences based on genetic distance by the Kimura 2-parameter model. The numbers on the branches are percent bootstrapping values from 1000 replicates. Each sequence is identified by its accession number, host origin, and genotype designation. The *E. bieneusi* genotype CSK2 (KY706128) from white kangaroo was used as the outgroup. The squares and triangles filled in black indicate novel and known genotypes identified in this study, respectively.

Based on the phylogenetic analysis of the neighbor-joining tree of the ITS gene sequences of *E. bieneusi*, all identified genotypes with the exception of genotype D, were in Group 2 (Fig. 3).

## Discussion

To date, there have been nine reports on the identification and genotyping of *E. bieneusi* in goats [1, 3, 4, 14, 16, 18, 21, 22, 28]. In this study, the prevalence of *E. bieneusi* in black goats was 24.0% (82/341) which was lower than that in Chongqing (62.5%; 5/8) [21] and Henan (31.2%; 186/596) [14, 18, 21], but higher than that in Anhui (5.1%; 33/654) [14, 21], Yunnan (12.8%; 60/470) [4, 21], Jiangsu (2.7%; 2/74) [14], Heilongjiang (21.8%; 12/55) [28], Shaanxi (19.9%; 72/361) [18, 21] and Tibet (9.6%; 25/260) [3]. Elsewhere, infection rates of 14.3% (1/7), 13.3% (11/83), and 19.2% (14/73) have been reported in goats from Spain [16], Egypt [1] and Thailand [22], respectively. The results suggested that goats are important hosts of *E. bieneusi* and may thus play a key role in the transmission of microsporidiosis caused by *E. bieneusi*.

In terms of age groups, we observed a significantly higher infection rate of *E. bieneusi* in kids (38.4%; 28/73) than in older goats (20.1%; 54/268) in Hainan (*χ*^2^ = 10.413, *p* < 0.01), which was consistent with previous findings [18, 21]. A recent study showed that the prevalence of *E. bieneusi* decreased with increasing age [4]. The high risk of younger goats to infection with *E. bieneusi* can be explained through their lower immune status and higher disease susceptibility.

To date, 46 genotypes of *E. bieneusi* have been identified in goats worldwide, 11 of which have been detected in humans [1, 3, 4, 14, 16, 18, 21, 22, 28]. In this study, eight genotypes were identified in goats, including six known genotypes (AHG1, CHG2, CHG3, CHG5, CM21 and D), and two novel genotypes (HNG-I and HNG-II). Out of the eight genotypes, genotype CHG5 was the most prevalent, with a prevalence of 57.3% (47/82), followed by CHG3 which had a prevalence of 28.0% (23/82) and then the remaining six genotypes with a lower prevalence of 14.6% (12/82). These results were different from those observed in other provinces of China. For example, the dominant genotypes found in Tibet were EbpC and EbpA, in Shaanxi genotype SX1, in Henan and Yunnan genotype BEB6, and in Anhui genotype CHG3. Therefore, *E. bieneusi* infections in goats likely differ by region.

All the studies on *E. bieneusi* in goats found at least one zoonotic genotype (BEB6, Peru6, EbpA and EbpC) with a high prevalence, except for the study from Anhui Province. Interestingly, Peru6 and EbpC were commonly found in humans from Yunnan and Heilongjiang Provinces; they were also found in animals including pigs, minks and birds [5, 7, 15, 27, 29]. Zoonotic genotype BEB6 was found in a child from Shanghai City, and it seems more common in herbivore animals in China, including deer, sheep, goats, cattle and horses [30]. These findings suggest that goats can transmit the above-mentioned zoonotic genotypes to humans and other animals. However, we did not identify genotypes BEB6, Peru6, EbpA and EbpC, but we found genotype D, which is responsible for the majority of human infections and has been found in humans in more than 40 countries or areas [13, 17]. In China, this genotype has been found in AIDS patients, cancer patients, children, and HIV-positive patients from Henan, Shanghai, Hubei, and Heilongjiang Provinces, summarized by Li et al. [13]. It has also been reported as the dominant genotype in no-human primates, horses, pigs, and cats, as well as in urban wastewater [1, 9–11, 23, 24, 27]. These results illustrate that the interspecies transmission of genotype D poses a high zoonotic risk, representing a public health concern within the human population.

The other five known genotypes (AHG1, CHG2, CHG3, CHG5 and CM21) identified were found in sheep or goats in previous studies [14, 18, 21]. Genotypes CHG2 and CHG3 have also been detected in dairy cattle [4, 12], and genotype CM21 was found in captive golden snub-nosed monkeys [25]. However, genotypes CHG5 and AHG1 were only found in goats [14, 18, 21]. To date, the potential of five known genotypes identified and the two novel genotypes (HNG-I and HNG-II) to cause disease in humans or other livestock is unknown. Their host adaptation and potential role in the zoonotic transmission of *E. bieneusi* infection now requires further study in more systematic molecular epidemiological investigations of *E. bieneusi* in a larger number of hosts.

## Conclusion

In conclusion, this is the first report on the identification of *E. bieneusi* from farmed black goats in Hainan Province, with high prevalence and wide occurrence demonstrated. It is necessary to develop improved farm management to prevent the occurrence of cross-transmission and re-infection of *E. bieneusi* between different individuals within each goat farm. Although the six known genotypes identified here have already been reported in goats, two novel genotypes were identified and provide novel insights into the genotypic variation in *E. bieneusi*. However, due to the small percentage of potentially zoonotic genotypes in these animals, there is a minimal risk of zoonotic transmission of *E. bieneusi*.
